# LncRNA TTN-AS1 regulates osteosarcoma cell apoptosis and drug resistance via the miR-134-5p/MBTD1 axis

**DOI:** 10.18632/aging.102325

**Published:** 2019-10-10

**Authors:** Dong Fu, Chunwen Lu, Xingzhou Qu, Peng Li, Kai Chen, Liancheng Shan, Xiaodong Zhu

**Affiliations:** 1 Children’s Hospital of Shanghai, School of Medicine, Shanghai Jiao Tong University, Shanghai, China; 2 Department of Spinal Surgery, Changhai Hospital, Affiliated to Naval Military Medical University, Shanghai, China; 3 The Department of Oral Maxillofacial and Head Neck Oncology in the Ninth Hospital Affiliated Shanghai Jiaotong University, Shanghai, China; 4 Hai’an People’s Hospital, Hai’an, Nantong, Jiangsu, China; 5 Department of Orthopedics, Tongren Hospital, Shanghai Jiao Tong University School of Medicine, Shanghai, China

**Keywords:** osteosarcoma, lncRNA TTN-AS1, miR-134-5p, malignant brain tumour domain containing protein 1, resistance, aging and age-related diseases

## Abstract

Aim: To explore the mechanism by which long non-coding RNA (lncRNA) TTN-AS1 regulates osteosarcoma cell apoptosis and drug resistance via the microRNA miR-134-5p/malignant brain tumour domain containing 1 (MBTD1) axis.

Results: The lncRNA TTN-AS1 was highly expressed in osteosarcoma and was associated with poor prognosis. The lncRNA TTN-AS1 promoted cell viability and inhibited apoptosis. MiR-134-5p targeted MBTD1, which was regulated by lncRNA TTN-AS1. MBTD1 was highly expressed in osteosarcoma and was associated with poor prognosis. MBTD1 promoted cell viability and inhibited apoptosis, and knockdown of MBTD1 reversed the cancer-promoting effects of lncRNA TTN-AS1. Downregulation of lncRNA TTN-AS1 reduced drug resistance.

Conclusion: In osteosarcoma, lncRNA TTN-AS1 promoted the expression of MBTD1 by targeting miR-134-5p and regulated cell growth, apoptosis and drug resistance.

Methods: The expression characteristics of genes in osteosarcoma patients were analysed using bioinformatics. Plasmid transfection technology was applied to silence or overexpress lncRNA TTN-AS1, miR-134-5p and MBTD1. Western blotting and quantitative polymerase chain reaction (qPCR) were used to detect protein and RNA, respectively. A cell counting kit 8 (CCK-8) and flow cytometry were used to detect cell viability and apoptosis. The effects of lncRNA TTN-AS1 and MBTD1 on osteosarcoma in vivo were studied by using a tumour burden assay.

## INTRODUCTION

Osteosarcoma is a musculoskeletal malignancy that originates in the interstitial cell line [1]. The incidence of osteosarcoma is approximately 3 per million, and the incidence of osteosarcoma in men is higher than that in women [2]. The predilection site of osteosarcoma is the medullary ends of the long bones, especially the distal femur and the proximal humerus around the knee joint [3]. The course of osteosarcoma progresses rapidly, and morbidity and mortality are high [4, 5]. Currently, the main treatments for osteosarcoma include surgery, chemotherapy, immunotherapy and gene therapy [6–8]. The strong proliferative capacity and drug resistance of osteosarcoma cells are important factors affecting prognosis [9, 10].

Long non-coding RNA (lncRNA) typically has a length of more than 200 nucleotides [11]. LncRNAs do not have protein translation capabilities, but they play an important role in various processes by directly or indirectly regulating protein expression [12]. LncRNAs can not only directly participate in the regulation of gene expression but can also act as competing endogenous RNAs (ceRNAs) to interact with miRNAs [13, 14]. A microRNA (miRNA) is an evolutionarily conserved single-stranded RNA that typically contains 21–24 nucleotides [15]. MiRNAs play a key role in the post-transcriptional regulation of mRNA by targeting the 3′ untranslated region (UTR) of mRNA, leading to mRNA degradation or translational inhibition [15]. In recent years, many studies have found that lncRNA is involved in the development and progression of osteosarcoma as ceRNA [16–18]. The number of single lncRNAs is large, and the pathways are diverse. Bioinformatics helps to screen for meaningful lncRNAs [19, 20].

In this paper, we found that the lncRNA TTN-AS1 and malignant brain tumour domain containing protein 1 (MBTD1) were overexpressed in osteosarcoma, and they were associated with the poor prognosis of osteosarcoma patients. Based on this, we revealed a novel mechanism by which lncRNA TTN-AS1 regulated proliferation and apoptosis of osteosarcoma cells via miR-134-5p/MBTD1. This may provide new ideas for the discovery of strategies for osteosarcoma treatment.

## RESULTS

### LncRNA TTN-AS1 is highly expressed in osteosarcoma and is associated with poor prognosis

**Figure 1:**
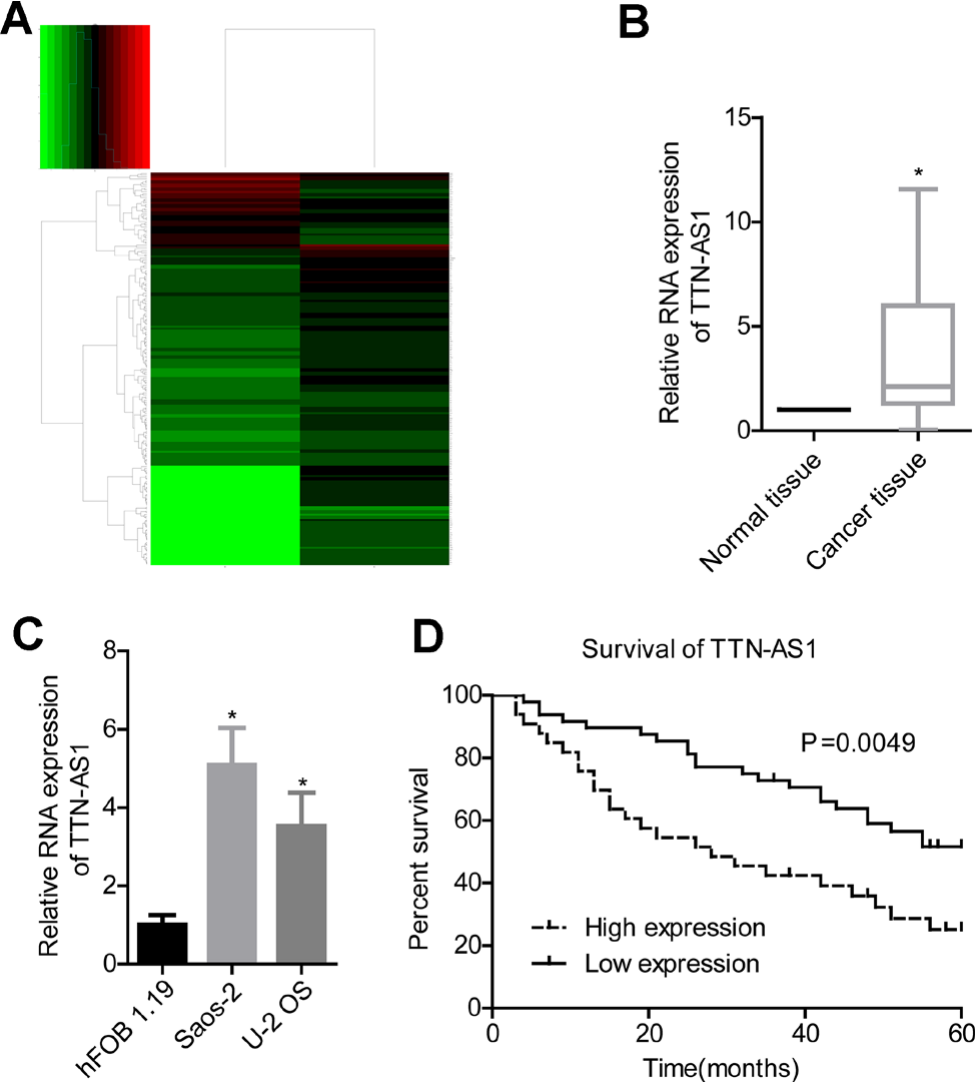
**lncRNA TTN-AS1 is highly expressed in osteosarcoma and is associated with poor prognosis.** (**A**) Bioinformatics analysis of differentially expressed genes in osteosarcoma. (**B**) QPCR was used to detect the expression level of lncRNA TTN-AS1 in tumour tissues and adjacent tissues. (**C**) QPCR was applied to detect lncRNA TTN-AS1 levels in osteosarcoma cell lines. (**D**) 5-year survival analysis was done on osteosarcoma patients with different lncRNA TTN-AS1 levels. ^*^P < 0.05 vs. normal tissue or hFOB 1.19 cell group;

Bioinformatics analysis revealed that lncRNA TTN-AS1 was upregulated in osteosarcoma (Figure 1A). Clinical sample test results also showed that lncRNA TTN-AS1 was highly expressed in tumour tissues (2.08 ± 2.45 vs. 1.00 ± 0.02) (Figure 1B). Overexpression of lncRNA TTN-AS1 was also present in osteosarcoma cell lines (5.09 ± 0.94 and 3.47 ± 1.04 vs. 1.00 ± 0.14) (Figure 1C). Patients with high expression of lncRNA TTN-AS1 had lower survival rates (Figure 1D). Further clinical analysis also showed that a high level of lncRNA TTN-AS1 was associated with higher Enneking stage and remote transfer (Table 1).

**Table 1:** Correlation between TTN-AS1 expression and clinicopathological features in OS patients.

**Characteristics**	**Total number**	**TTN-AS1 expression**		***P* value**
		**Low (n=29)**	**High (n=26)**	
Age (years)				0.651
<18	11	4	7	
≥18	44	25	19	
Gender				0.090
Male	36	16	20	
Female	19	13	6	
Tumor site				0.385
Femur/ Tibia	18	11	7	
Other	37	18	19	
Enneking stage				0.041
I-II-A	39	24	15	
II-B/IVB	16	5	11	
Lymph node metastasis				0.014
Absent	47	28	19	
Present	8	1	7	

### LncRNA TTN-AS1 promotes cell viability and inhibits apoptosis

**Figure 2:**
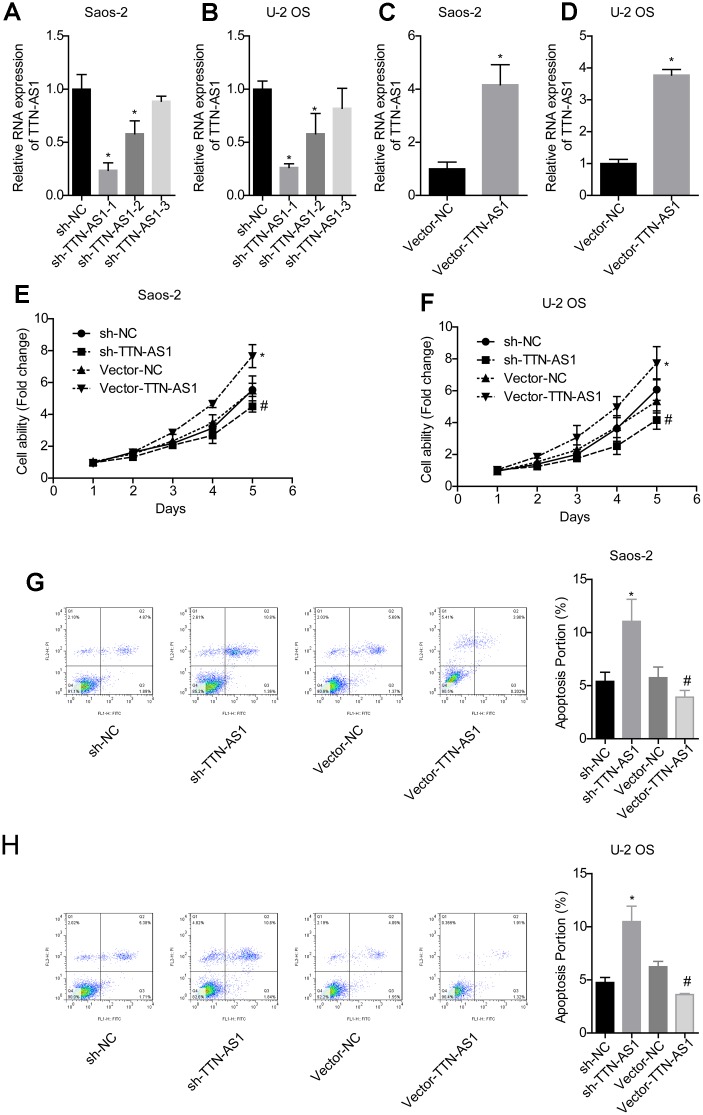
**lncRNA TTN-AS1 promoted cell viability and inhibited apoptosis.** (**A**–**D**) QPCR was used to detect the expression level of lncRNA TTN-AS1. (**E**, **F**) The cell viability of each group of cells was detected by the CCK-8 method. (**G**, **H**) The apoptosis rate of each group of cells was tested using the CCK-8 method. ^*^P < 0.05 vs. sh-NC group; ^#^P < 0.05 vs. vector-NC.

QPCR results showed that the TTN-AS1 level in the sh-TTN-AS1-1 and sh-TTN-AS1-2 groups was lower than that in the sh-NC group (Saos-2: 0.24 ± 0.08 and 0.55 ± 0.17 vs. 1.00 ± 0.19; U-2OS: 0.25 ± 0.04 and 0.54 ± 0.23 vs. 1.00 ± 0.10), while vector-TTN-AS1 significantly upregulated the level of TTN-AS1 (Saos-2: 4.06 ± 0.92 vs. 1.00 ± 0.09; U-2OS: 4.25 ± 0.89 vs. 1.00 ± 0.06), indicating successful transfection (Figure 2A–2D). Sh-TTN-AS1-1 was used for subsequent experiments and referred to as sh-TTN-AS1. The relative cell viability in the vector-TTN-AS1 group (Saos-2: 7.24 ± 0.35; U-2OS: 7.60 ± 0.47) was significantly higher than that of the vector-NC group (Saos-2: 5.28 ± 0.31; U-2OS: 5.88 ± 0.28). In addition, the relative cell viability in the sh-TTN-AS1 group (Saos-2: 4.08 ± 0.11; U-2OS: 4.01 ± 0.15) was lower than that of the sh-NC group (Saos-2: 5.02 ± 0.13; U-2OS: 1.13 ± 0.14) (Figure 2E–2F). The apoptosis rate was increased after transfection with sh-TTN-AS1 (Saos-2: 11.04 ± 2.44%; U-2OS: 10.49 ± 0.20%), and the apoptosis rate was decreased after transfection with vector-TTN-AS1 (Saos-2: 3.92 ± 0.08%; U-2OS: 3.62 ± 0.04%) (Figure 2G–2H). This indicated that lncRNA TTN-AS1 promoted cell viability and inhibited apoptosis.

### LncRNA TTN-AS1 targets miR-134-5p

**Figure 3:**
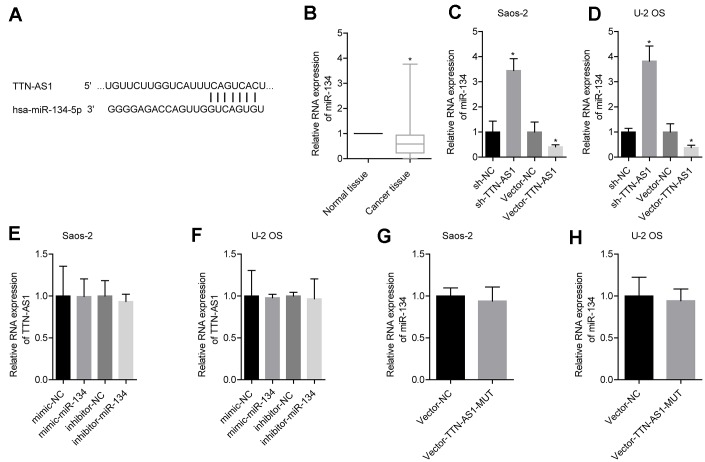
**lncRNA TTN-AS1 targeted miR-134-5p.** (**A**) The website predicted that lncRNA TTN-AS1 targeted miR-134-5p. (**B**) QPCR was used to detect the expression level of miR-134-5p in tumour tissues and adjacent tissues. (**C**, **D**) The effects of downregulation or upregulation of lncRNA TTN-AS1 on miR-134-5p are shown. (**E**, **F**) The effects of downregulation or upregulation of miR-134-5p on lncRNA TTN-AS1 are shown. (**G**, **H**) The effects of transfected mutant lncRNA TTN-AS1 on miR-134-5p are shown. ^*^P < 0.05 vs. normal tissue or vector-NC.

The predicted results from the bioinformatics analysis showed that lncRNA TTN-AS1 targeted miR-134-5p (Figure 3A). Clinical sample test results showed that miR-134-5p was expressed at low levels in osteosarcoma (0.52 ± 0.41) (Figure 3B). Further experimental results showed that upregulation of lncRNA TTN-AS1 inhibited the expression of miR-134-5p, and downregulation of lncRNA TTN-AS1 gave the opposite results (Figure 3C–3D). Promotion or inhibition of miR-134-5p expression had no significant effects on lncRNA TTN-AS1 (Figure 3E–3F). Furthermore, the mutated lncRNA TTN-AS1 did not cause changes in the level of miR-134-5p (Figure 3G–3H). This indicated that lncRNA TTN-AS1 could be a target to regulate the level of miR-134-5p.

### MiR-134-5p targets MBTD1, which is regulated by lncRNA TTN-AS1

**Figure 4:**
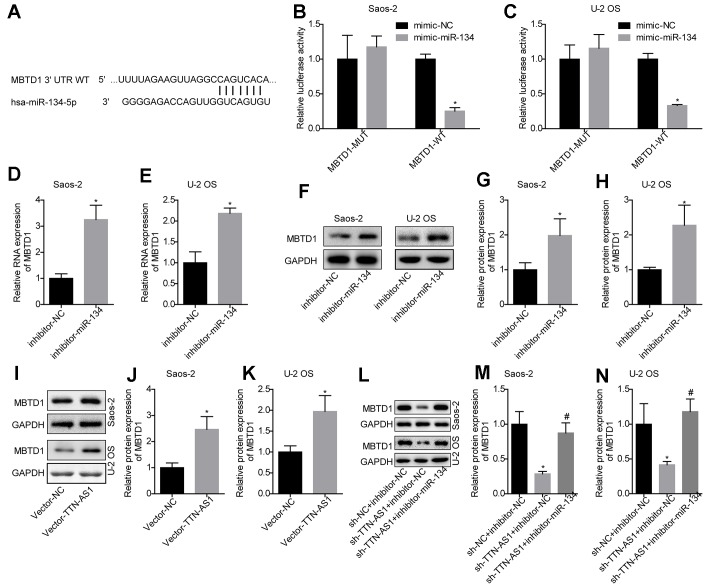
**MiR-134-5p targets MBTD1, which is regulated by lncRNA TTN-AS1.** (**A**) The website predicted that MBTD1 was the target gene of miR-134-5p. (**B**, **C**) The luciferase assay confirmed that miR-134-5p targets MBTD1. (**D**–**H**) The effects of down-regulating miR-134-5p on MBTD1 mRNA and protein expression levels are shown. (**I**–**K**) The effects of upregulation of lncRNA TTN-AS1 on MBTD1 protein expression are shown. (**L**–**N**) The effects of knockdown of lncRNA TTN-AS1 and miR-134-5p on MBTD1 protein expression are shown. ^*^P < 0.05 vs. mimic-NC, inhibitor-NC or sh-NC + inhibitor-NC group; ^#^P < 0.05 vs. sh-TTN-AS1 + inhibitor-NC.

The TargetScan website was used to predict the target gene of miR-134-5p (Figure 4A). The luciferase assay demonstrated that miR-134-5p targeted the 3′-UTR region of MBTD1 (Figure 4B, 4C). Further studies also showed that compared with the inhibitor-NC group, inhibition of miR-134-5p levels could upregulate MBTD1 mRNA and protein levels (Saos-2: 3.14 ± 0.67 and 1.98 ± 0.55; U-2OS: 2.21 ± 0.13 and 2.07 ± 0.57) (Figure 4D–4H). This indicated that miR-134-5p can directly target MBTD1.

The results also showed that upregulation of lncRNA TTN-AS1 promoted MBTD1 protein expression (Saos-2: 2.27 ± 0.44; U-2OS: 1.98 ± 0.48) (Figure 4I–4K). In addition, the results showed that downregulation of lncRNA TTN-AS1 levels inhibited MBTD1 protein expression (Saos-2: 0.26 ± 0.03; U-2OS: 0.38 ± 0.03), while inhibitors partially reversed the inhibitory effects of lncRNA TTN-AS1 on MBTD1 (Saos-2: 0.82 ± 0.41; U-2OS: 1.17 ± 0.42) (Figure 4L–4N). This suggested that lncRNA TTN-AS1 could regulate the expression level of MBTD1 by targeting miR-134-5p.

### MBTD1 is highly expressed in osteosarcoma and is associated with poor prognosis, and MBTD1 promotes cell viability and inhibits apoptosis

**Figure 5:**
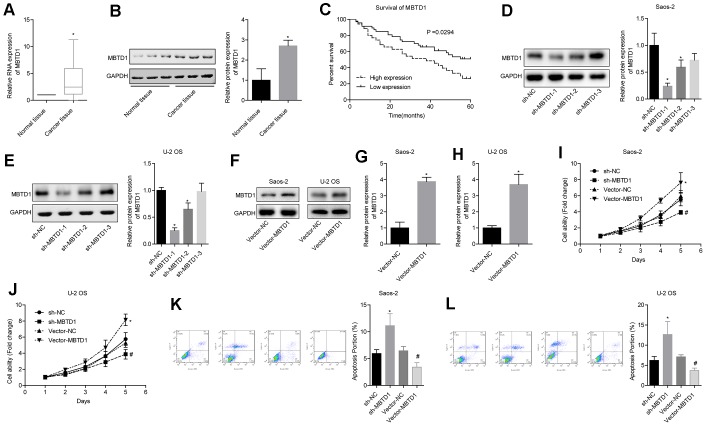
**MBTD1 is highly expressed in osteosarcoma and associated with poor prognosis, and MBTD1 promotes cell viability and inhibits apoptosis.** (**A**, **B**) The expression level of MBTD1 in osteosarcoma tissues and adjacent tissues is shown. © 5-year survival analysis of osteosarcoma patients with different MBTD1 levels is shown. (**D**–**H**) The effects of MBTD1 knockdown or overexpression on MBTD1 protein expression levels are shown. (**I**–**L**) The effect of knockdown or overexpression of MBTD1 on cell viability and apoptosis is shown. ^*^P < 0.05 vs. normal tissue or sh-NC group; ^#^P < 0.05 vs. vector-NC.

The level of MBTD1 in cancer tissues was significantly higher than that in normal tissues (Figure 5A, 5B). Osteosarcoma patients with high expression of MBTD1 had worse 5-year survival rates compared to those with low expression of MBTD1 (50.005 vs. 26.43%) (Figure 5C). Western blotting results showed that MBTD1 protein levels in the sh-MBTD1-1 and sh-MBTD1-2 groups were significantly lower than those in the sh-NC group, indicating successful transfection experiments (Figure 5D–5E). sh-MBTD1-1 was used for subsequent experiments and referred to as sh-MBTD1. Cell lines overexpressing MBTD1 were also successfully established, and MBTD1 protein levels were upregulated 3.85 (± 0.38) and 3.67 (± 0.72) times in Saos-2 and U-2OS, respectively (Figure 5G–5H). Further studies showed that overexpression of MBTD1 promoted cell viability and inhibited apoptosis, while downregulation of MBTD1 inhibited cell viability and promoted apoptosis (Figure 5G–5L). This suggested that MBTD1 had a role in promoting tumour growth.

### LncRNA TTN-AS1 inhibited tumour growth via MBTD1.

**Figure 6:**
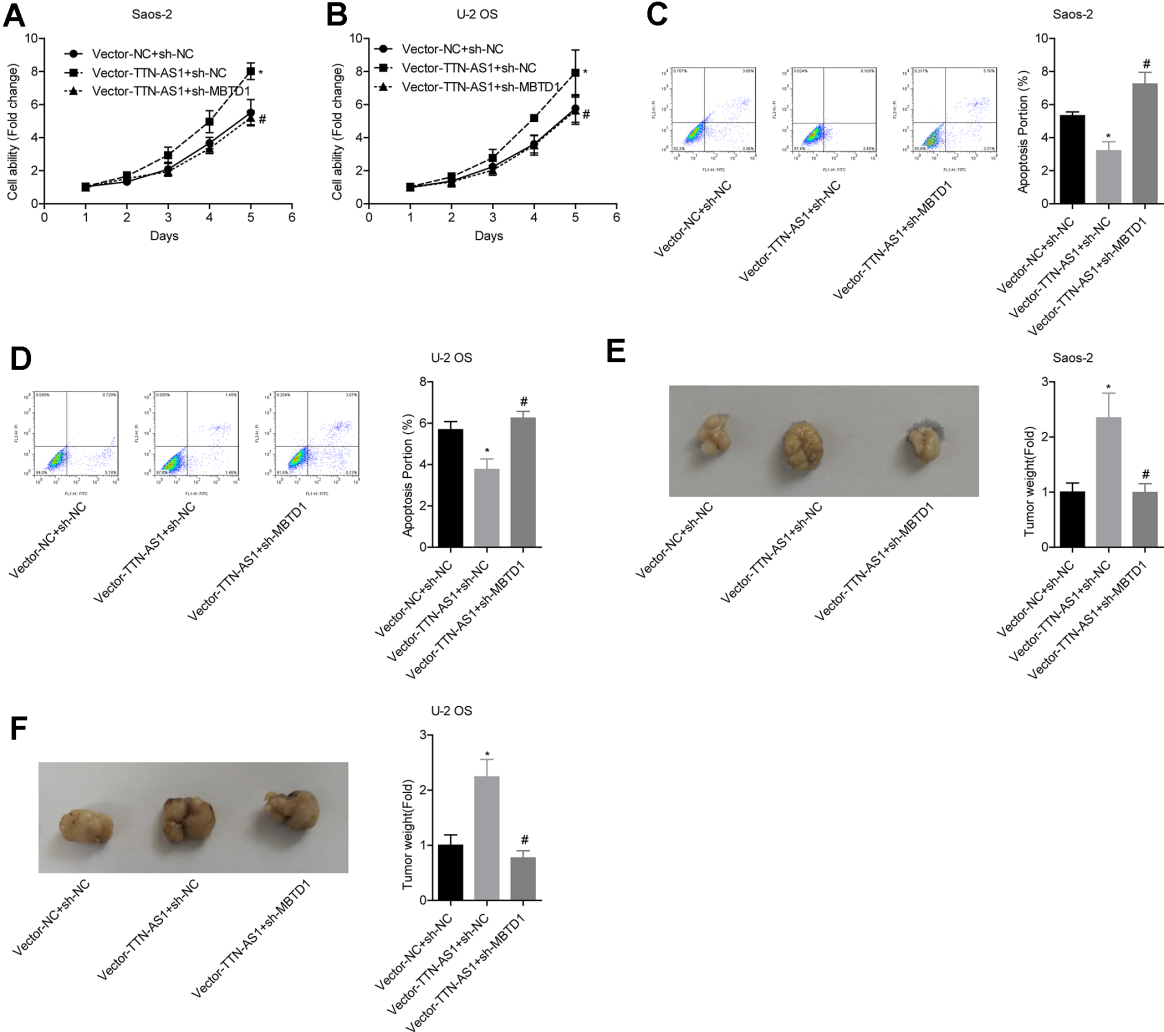
**lncRNA TTN-AS1 inhibits tumour growth via MBTD1.** (**A**–**D**) The effects of overexpression of lncRNA TTN-AS1 and knockdown of MBTD1 on cell viability and apoptosis are shown. (**E**, **F**) The effects of overexpression of lncRNA TTN-AS1 and knockdown of MBTD1 on tumour growth are shown. ^*^P < 0.05 vs. vector-NC + sh-NC group; ^#^P < 0.05 vs. vector-TTN-AS1 + sh-NC.

The relative cell viability of the vector-TTN-AS1 + sh-NC group (Saos-2: 7.95 ± 0.27; U-2OS: 7.39 ± 0.66) was significantly higher than that of the vector-NC group; the cell viability of the Vector-TTN-AS1 + sh-MBTD1 group (Saos-2: 4.92 ± 0.18; U-2OS: 4.20 ± 0.31) was lower than that of the Vector-TTN-AS1 + sh-NC group (Figure 6A–6B). The relative apoptosis rate of the Vector-TTN-AS1 + sh-NC group (Saos-2: 3.17 ± 0.62; U-2OS: 3.72 ± 0.48) was significantly lower than that of the vector-NC group; the cell viability of the Vector-TTN-AS1 + sh-MBTD1 group (Saos-2: 7.11 ± 0.64; U-2OS: 6.08 ± 0.40) was higher than that of the Vector-TTN-AS1 + sh-NC group (Figure 6C–6D). These results suggested that knockdown of MBTD1 reversed the cancer-promoting effects of overexpression of lncRNA TTN-AS1. Animal experiments showed that the tumour weight of the vector-TTN-AS1 + sh-NC group (Saos-2: 2.66 ± 0.69; U-2OS: 2.24 ± 0.43) was significantly higher than that of the vector-NC group; the cell viability of the vector-TTN-AS1 + sh-MBTD1 group (Saos-2: 0.99 ± 0.18; U-2OS: 0.76 ± 0.11) was lower than that of the Vector-TTN-AS1 + sh-NC group (Figure 6E–6F).

### Downregulation of lncRNA TTN-AS1 reduces drug resistance

**Figure 7:**
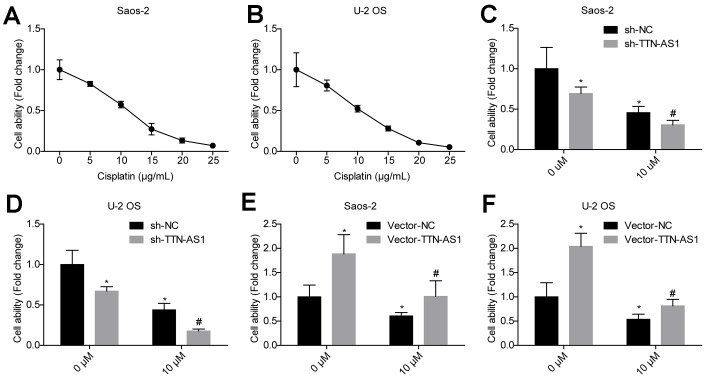
**Downregulation of lncRNA TTN-AS1 reduces drug resistance.** (**A**, **B**) The effects of different concentrations of cisplatin on the viability of osteosarcoma cells are shown. (**C**, **D**) Downregulation of lncRNA TTN-AS1 enhanced the inhibitory effects of cisplatin on osteosarcoma cells. (**E**, **F**) Upregulation of lncRNA TTN-AS1 attenuated the inhibitory effect of cisplatin on osteosarcoma cells. ^*^P < 0.05 vs. sh-NC and vector-NC group of 0 μM; ^#^P < 0.05 vs. sh-NC and vector-NC group of 10 μM.

The cell viability of the osteosarcoma cell lines Saos-2 and U2OS treated with different concentrations of cisplatin was examined. The cell viability decreased by approximately 50% when the concentration of cisplatin was 10 μM (Figure 7A–7B). The relative cell viability of the sh-TTN-AS1 group (Saos-2: 0.27 ± 0.02; U-2OS: 0.15 ± 0.01) was significantly lower than that of the sh-NC group (Saos-2: 0.46 ± 0.04; U-2OS: 0.45 ± 0.05) in the presence of 10 μM cisplatin (Figure 7C–7D). The relative cell viability of the vector-TTN-AS1 group (Saos-2: 0.96 ± 0.47; U-2OS: 0.78 ± 0.30) was significantly higher than that of the sh-NC group (Saos-2: 0.75 ± 0.02; U-2OS: 0.51 ± 0.03) in the presence of 10 μM cisplatin (Figure 7E–7F). Upregulation of lncRNA TTN-AS1 partially reverses the inhibitory effect of cisplatin on cells.

## DISCUSSION

In this study, we discovered the role of lncRNA TTN-AS1 and MBTD1 in osteosarcoma and analysed the mechanism by which lncRNA TTN-AS1 regulates apoptosis and drug resistance of osteosarcoma cells through the miR-134-5p targeting axis.

The role of lncRNA in osteosarcoma has been confirmed, and both lncRNA and miRNA can be used as markers for the diagnosis and prognosis of osteosarcoma [21–23]. This study found that lncRNA TTN-AS1 was highly expressed in osteosarcoma patients and was associated with poor prognosis. TTN-AS1 is located on chromosome 2: 178,521,183e178,779,963 and was first discovered to be associated with oesophageal squamous cell carcinoma. Patients with high expression of lncRNA TTN-AS1 have a worse prognosis and a higher risk of metastasis [24]. The results of Luo’s study [25] also show that lncRNA TTN-AS1 is overexpressed in lung adenocarcinoma and promotes tumour development by modulating the PTEN/PI3K/AKT pathway. Recent studies show that in cervical cancer, gastric cancer and thyroid cancer, lncRNA TTN-AS1 can act as a ceRNA to promote cancer by targeting miRNA [26–28]. However, the mechanism of action of lncRNA TTN-AS1 in tumours is unclear, and there is no research on lncRNA TTN-AS1 in osteosarcoma. The results of this study showed that lncRNA TTN-AS1 could promote cell viability and inhibit apoptosis, and downregulation of lncRNA TTN-AS1 could reduce drug resistance. This revealed the pro-cancer effects of lncRNA TTN-AS1 on osteosarcoma and the potential for involvement in osteosarcoma drug resistance.

To further explore the mechanism by which lncRNA TTN-AS1 regulated osteosarcoma cell growth and apoptosis, we found through bioinformatics prediction and experimental studies that lncRNA TTN-AS1 targeted miR-134-5p levels as ceRNA and that miR-134-5p inhibited MBTD1 expression by directly binding to the 3′UTR region of MBTD1 mRNA. Studies have found anti-tumour mechanisms of miR-134-5p in non-small cell lung cancer and nasopharyngeal carcinoma [29–31]. MiR-134-5p can also be targeted and regulated by lncRNA [32, 33]. Studies have found that miR-134-5p exerts anti-tumour effects by affecting target gene expression in osteosarcoma [34–36]. This study demonstrated that miR-134-5p was regulated by lncRNA TTN-AS1 to inhibit osteosarcoma growth and apoptosis.

MBTD1 belongs to the polycomb group (PcG) and regulates the transcription process. This study found the role of MBTD1 in tumourigenesis [37]. MBTD1 is associated with low-grade endometrial cancer [38]. However, the role of MBTD1 in osteosarcoma has not been studied. In this study, MBTD1 was highly expressed in osteosarcoma, and high levels of MBTD1 were closely related to malignancy and poor prognosis of osteosarcoma. Cell experiments confirmed that MBTD1 could promote cell viability and inhibit apoptosis. Both the cell experiments and animal experiments confirmed that downregulating the level of MBTD1 reversed the effects of overexpression of lncRNA TTN-AS1 on tumour growth.

In conclusion, lncRNA TTN-AS1 promoted the expression of MBTD1 by targeting miR-134-5p and regulated cell growth, apoptosis and drug resistance in osteosarcoma. However, further research is needed on the mechanisms by which TTN-AS1 participates in drug resistance and by which MBTD1 promotes the development of osteosarcoma.

## MATERIALS AND METHODS

### Bioinformatics analysis

The osteosarcoma patient gene data (GSE12865) were downloaded from the GEO database (https://www.ncbi.nlm.nih.gov/geo/). A difference analysis was performed on the data. The miRcode website (http://www.mircode.org/) performed a biosignal analysis to predict miRNAs downstream of lncRNA TTN-AS1. The TargetScan website (http://www.targetscan.org/vert_72/) was used to predict downstream target proteins of miR-134-5p.

### Osteosarcoma tissues and research

55 osteosarcoma tissues and 9 adjacent tissues were purchased from Alenabio Biotechnology Co., Ltd. (OS208a, Xi’an, China). Quantitative polymerase chain reaction (qPCR) was used to detect lncRNA TTN-AS1, miR-134-5p and MBTD1 mRNA expression levels in cancer tissues and normal tissues. The 5-year survival of patients with high or low expression of lncRNA TTN-AS1 and MBTD1 mRNA was compared. The correlation between lncRNA TTN-AS1 and pathological features of patients with osteosarcoma was analysed. Approval was obtained from the Tongren Hospital Ethics Committee.

### Cells culture and transfection

Human osteoblast hFOB 1.19 and osteosarcoma cell lines Saos-2 and U-2OS were purchased from American Type Culture Collection (Manassas, VA, USA) and were grown in RPMI-1640 medium containing 10% foetal bovine serum (FBS, Thermo Fisher, Waltham, USA), 50 U/mL penicillin and 50 μg/mL streptomycin (15070063, Thermo Fisher, Waltham, USA). The plasmid constructs and the corresponding negative control (NC) were built by Shanghai Genepharma Company (China). Plasmid transfection technology was applied to silence or overexpress lncRNA TTN-AS1, miR-134-5p and MBTD1. The cells were transfected with 100 nM RNA oligonucleotides using LipofectamineTM 2000 (Invitrogen, Waltham, USA). Cells were harvested 48 h after transfection for subsequent experiments.

### QPCR

Total RNA was acquired using TRIzol (Invitrogen, USA). The cDNA was generated from 1 μg isolated miRNA/mRNA by using an iScriptTMcDNA Synthesis Kit (Bio-Rad, USA). For qPCR processing, the FastStart Universal SYBR Green Master kit (Roche, Switzerland) was used. The reaction system was prepared as follows: 2.5 μL cDNA template, 1 μL forward primer (10 μM), 1 μL reverse primer (10 μM), 10 μL 2 × SYBR Green master mix, 5.5 μL dH_2_O. QPCR was performed using the following procedure: 2 min at 95°C, 40 cycles of 15 sec at 95°C, 25 sec at 60°C and 60 sec at 72°C. U6 acted as an internal reference for miR-134-5p, and GAPDH served as an internal reference for lncRNA and mRNA. The primers were synthesized by Sangon Biotech (Shanghai, China) and are shown in Table 2.

**Table 2:** Primers for qPCR.

**Genes**	**Forward (5′-3′)**	**Reverse (5′-3′)**
TTN-AS1	CCAGACACCTAACCAACTTCC	GTGATCTCATCCCTCTTGCTT
miR-134-5p	GAAGCTCATTGGAGACCCTAAC	CAACCTCTAAGTCGTGCTCATAC
MBTD1	CTACAGCCTCCAGCATCACA	CTCATCAGCTGACCCAGACA
GAPDH	AGAAGGCTGGGGCTCATTTG	AGGGGCCATCCACAGTCTTC
U6	CTCGCTTCGGCAGCACA	AACGCTTCACGAATTTGCGT

### Western blot analysis

The whole-cell protein was extracted by using ice-cold RIPA buffer mixed with protease inhibitors. The protein lysate was separated by SDS-PAGE at 110 V for 100 min and transferred to PVDF membranes at 90 V for 90 min. The PVDF membrane was blocked in 5% non-fat milk for 1 h at room temperature, and antibody (MBTD1, ab170848, Abcam, USA, 71 kD; GAPDH, ab9484, 36 kD) was added at 4°C overnight. The membrane was further probed with the secondary antibody IgG H&L (HRP) (ab6721, Abcam, San Francisco, USA, 1:5000) after washing the membrane with PBST (PBS with 0.2% Tween 20). The protein bands were detected by Pierce ECL Plus western blotting substrate (Thermo Fisher, Waltham, USA) on a ChemiDoc MP instrument (Bio-Rad, California, USA).

### Cell counting kit 8 (CCK-8) assay

The cells were seeded in a 96-well culture plate (2 × 10^4^ cells / mL, 100 μL / well), and the blank controls (with medium alone) were set. After hypoxia-reoxygenation, 10 μL of CCK-8 (Beyotime Institute of Biotechnology, Beijing, China) reagent was added to each well, and the culture was continued for 2 h at 37°C. The absorbance values were measured at 450 nm on a microplate reader (Tecan Infinite M200 Micro Plate Reader; LabX, Switzerland), and the corresponding optical density ratio was expressed as cell vitality.

### Cell apoptosis

The 2×10^5^ cells were collected in 1.5 mL EP tubes. The supernatant was discarded after centrifugation at 2000 × *g* at 4°C. Then, 500 μL of binding buffer was added to suspend the cells, and 5 μL of Annexin V-FITC was added and incubated at 4°C for 30 min in the dark. Then, 5 μL of propidium iodide (PI) was added, gently mixed and incubated at room temperature for 5 min. The cell apoptosis rates were detected using an Annexin-V-FITC detection kit (K201-100, BioVision, USA) and flow cytometry (version 10.0, FlowJo, FACS CaliburTM, BD, USA).

### Luciferase assay

The sequences containing the wild-type (WT) or mutated (Mut) region MBTD1 were synthesized by Sangon (Shanghai, China) and inserted into a pGL3 vector (Promega Corporation, Madison, USA). For the luciferase reporter assay, miR-134-5p mimics and the respective reporter plasmids were transfected into cells using Lipofectamine 2000 according to the manufacturer’s protocol. After 24 h, the Renilla and Firefly luciferase activities were determined using the Dual-Luciferase Reporter Assay System (Promega Corp.) and a luminometer (Infinite 200 PRO NanoQuant; Tecan Group Ltd., Männedorf, Switzerland).

### Tumour burden assay

Specific pathogen-free (SPF) 4-week-old BALB/c nude mice were purchased from the animal centre of Air Force Medical University (Shanghai, China). Saos-2 and U2OS cells were divided into three groups: vector-NC + sh-NC, vector-TTN-AS1 + sh-NC and vector-TTN-AS1 + sh-MBTD1. The mice in each group were injected with 1×10^6^ cells correspondingly. Twenty-eight days after injection, the mice were sacrificed, and tumours were removed to be weighed and photographed. The animal experiment protocol was approved by the Animal Experimentation Ethics Committee of Tongren Hospital.

### Statistical analysis

All analyses were conducted using Prism GraphPad 7.0 software. All variables were shown as the mean ± standard deviation (SD). Comparisons among multiple groups were performed using one-way analysis of variance (ANOVA). P < 0.05 was considered statistically significant.
